# Study of the betulin enriched birch bark extracts effects on human carcinoma cells and ear inflammation

**DOI:** 10.1186/1752-153X-6-137

**Published:** 2012-11-19

**Authors:** Cristina A Dehelean, Codruţa Şoica, Ionuţ Ledeţi, Mihaela Aluaş, Istvan Zupko, Atena Gǎluşcan, Simona Cinta-Pinzaru, Melania Munteanu

**Affiliations:** 1 Faculty of Pharmacy, Victor Babeş University of Medicine and Pharmacy, Eftimie Murgu Square 2, Timişoara, RO- 300041, România; 2 Faculty of Physics, Babes-Bolyai University, Kogalniceanu 1, Cluj-Napoca, 400084, Romania; 3 Department of Pharmacodynamics and Biopharmacy, University of Szeged, Szeged, Hungary; 4 Faculty of Dentistry, Victor Babes University of Medicine and Pharmacy Timisoara, 9th Revolutiei din 1989 Blvd, Timisoara, 300041, Romania; 5 Department of Clinical Laboratory and Sanitary Chemistry, “Vasile Goldis” University, 1 Feleacului Str, Arad, 310396, Romania

**Keywords:** Betulin, Birch extract, Anticancer, Raman, SERS, ^13^C NMR

## Abstract

**Background:**

Pentacyclic triterpenes, mainly betulin and betulinic acid, are valuable anticancer agents found in the bark of birch tree. This study evaluates birch bark extracts for the active principles composition.

**Results:**

New improved extraction methods were applied on the bark of *Betula pendula* in order to reach the maximum content in active principles. Extracts were analyzed by HPLC-MS, Raman, SERS and ^13^C NMR spectroscopy which revealed a very high yield of betulin (over 90%). Growth inhibiting effects were measured *in vitro* on four malignant human cell lines: A431 (skin epidermoid carcinoma), A2780 (ovarian carcinoma), HeLa (cervix adenocarcinoma) and MCF7 (breast adenocarcinoma), by means of MTT assay. All of the prepared bark extracts exerted a pronounced antiproliferative effect against human cancer cell lines. In vivo studies involved the anti-inflammatory effect of birch extracts on TPA-induced model of inflammation in mice.

**Conclusions:**

The research revealed the efficacy of the extraction procedures as well as the antiproliferative and anti-inflammatory effects of birch extracts.

## Background

Pentacyclic triterpenes are a class of compounds extensively studied as future anticancer agents. One of the most studied substances within this class is betulinic acid (BA) whose antineoplasic effectiveness is diminished by its poor water solubility [1]. That is why a lot of efforts have been put in surpassing this disadvantage by either obtaining complexes with hydrophilic substances such as cyclodextrins [2] or by derivatising BA to more soluble compounds [3].

**Figure 1:**
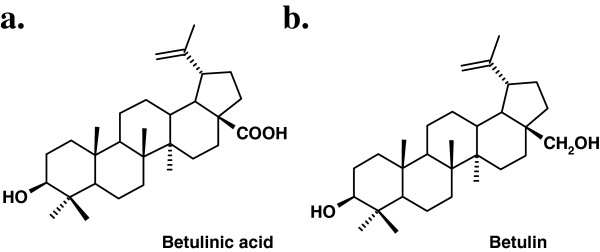
Betulinic acid (a) and betulin (b) structural formula.

Betulinic acid (Figure 1a) can be found in many plants, especially *Betula* sp.; also, it can be obtained by chemical synthesis from its precursor, betulin (Figure 1b), which is present in the same specie [1]. The percentage of active substances in the birch bark differs from one species to another; few studies have been made on *Betula pendula* Roth which can be found in Romania, regarding the content in betulin/betulinic acid in its outer bark.

Birch tree has been known for a long time for its healing properties; birch bark oil was used in folk medicine for the treatment of skin diseases (eczema, psoriasis) [4]. It is known that Native Americans used the bark to prepare teas for the treatment of digestive tract infections [5], so it is expected that the use of the above mentioned species as raw material for the extraction of active principles could be an important source of compounds for the treatment of various pathologies. *In vitro* and *in vivo* studies have revealed the interferon-inducing activity of both dry birch bark extract and betulin [6]. Betulin has three active positions in its structure, secondary hydroxyl group at C-3, primary hydroxyl group at C-28 and a double C-C bonding at C-20, where chemical modulations can be performed in order to obtain different types of derivatives [7]. By oxidation of the C-28 hydroxylic group to carboxyl, betulin can be rapidly converted to its more active derivate, betulinic acid, with a wide spectrum of biological and pharmacological activities such as: anti-inflammatory, antimalarial, anti-HIV, antineoplasic [8,9].

Recent studies have shown the possibility of obtaining an extract from the birch tree outer bark with over 70% betulin [10]. Our present work combines solvent extraction with a heating procedure in order to prepare a precipitate, based on the betulin’s sublimation property [11]. This combination of procedures led to a content of over 90% betulin in the dry extract.

## Results and discussion

The present report continues the work previously presented [12] by our team, falling in a project which focuses on finding new antitumor natural sources like birch bark; in the same time our present report brings important new research data. We improved in a unique manner the extraction procedure of betulin and betulinic acid from birch bark, by obtaining a higher amount of active compound (over 90%) than previous studies. Also, we enriched the knowledge on birch bark extract regarding its antitumor properties by analyzing its activity on a new cell line (A2780). All the elements are new and represent a step forward in the preclinical evaluation of these important antitumor compounds.

In the present HPLC/MS analysis the expected ion, according to betulin molecular weight (M = 442.3), would be its proton aduct, at m/z 443.3. Against expectations, this ion has not been noticed, but a majority ion was spotted at m/z 425.3, corresponding to the aduct formed with the proton by the dehydrated betulin molecule. Basically, betulin molecule dehydrates at the drying temperature of mobile phase inside the chromatograph (400°C) or during arc ionisation followed by protonation. Retention times were 2.75 min for betulin and 3 min for betulinic acid (Additional file 1: Figure S1).

HPLC technique has been previously used for the quantification of pentacyclic triterpenes in natural extracts [13]. In our previous study [12] application of HPLC/MS analysis revealed 57% percentage of betulin and 3% betulinic acid in the birch bark of *Betula pendula* Roth.

Isolation of triterpenes was reported from birch species; birch bark revealed the presence of triterpene derivates of lupane skeleton, like betulin and betulinic acid [14]. Betulin is an abundant natural triterpene, its presence in natural sources making the extraction procedure very important for large scale production. Extraction procedures are very different, from ultrasonic-assisted extraction [15] to supercritical carbon dioxide extraction [16].

The percentage of the active components in the birch bark differs from one specie to another; Diouf et al. [17] reported in 2009 a 56% betulin in the bark of yellow birch (*Betula alleghaniensis Britton*) in Quebec, Canada. A 34% content in betulin was reported by Jäger et al. in 2008 [18]. However, few studies have been made on the specie, *Betula pendula* Roth, in terms of the content of betulin/betulinic acid in the outer bark. Extraction of betulin can be conducted using high boiling point hydrocarbon solvents or water azeotrops of alcohols [19].

In a previous study [12] we used a mixture of solvents and continuous Soxhlet extraction for betulin preparation, reaching a percentage of over 50% active compound. In the present article we used two organic solvents, ethanol and propanol, with much improved results, up to 97% (see Table 1). This high amount of betulin makes this birch species very suitable for industrial preparation of betulin and its derivatives.

**Table 1:** Concentration of betulin / betulinic acid in the HPLC tested samples

**Sample**	**Weighed quantity**	**Active principle concentration in HPLC tested sample**		**Total quantity in the sample**		**% of active substance in sample**	
	**mg**	**ng/ml**		**mg**			
		**Betulinic acid**	**Betulin**	**Betulinic acid**	**Betulin**	**Betulinic acid**	**Betulin**
1	9.96	393.79	736.12	0.39	7.36	4.0	73.9
2	8.08	296.03	536.09	0.30	5.36	3.7	66.3
3	10.31	204.99	795.77	0.20	7.96	2.0	77.2
1 pp	10.47	256.61	852.12	0.26	8.52	2.5	81.4
3 pp	12.5	181.68	1216.79	0.18	12.17	1.5	**97.3**
4	8.12	135.90	628.50	0.14	6.28	1.7	77.4

^13^C-NMR spectroscopy is a highly sensitive technique that has been used for betulin characterization. Solid state ^13^C-NMR spectra were recorded for all prepared sample. From the integrated analysis of the betulin peaks and of the compound resonances in the region of 10–110 ppm we can conclude that the presence of betulin in the extracts is significantly higher compared to the previously reported products [12]. The presence of betulinic acid could not be determined from this experiment, which leads us to the conclusion that the amount of betulinic acid in the extract is smaller than 5%. Moreover, the samples 1 pp and 3 pp (Additional file 2: Figure S2) are characterized by a high degree of crystallinity revealed by the shapes of the solid-state NMR resonances.

A complete vibrational characterization of betulin was performed by our group and a few direct applications of this characterization were also previously presented [20,21].

Raman spectroscopy could provide semi-quantitative information concerning the triterpene content in the final extracts by analyzing the relative intensity ratio of the 1642 cm^-1^ (triterpene C = C mode) and 1601 cm^-1^ band, assigned to other organic residual compounds from the bark. Upon betulin vibrational characterization [21], we demonstrated the possibility to apply Raman spectroscopy for the prediction of the highest triterpene content in bark species, for selecting the appropriate harvesting period or for the direct selection in the field of individual genotypes using appropriate portable Raman equipment.

The relative intensity ratio of the band at 1642 cm^-1^ representative for the triterpenes and the band at 1606 cm^-1^ assigned to other species than triterpenes, I_1642_ / I_1606_ varied from 0.9 to 1.1 in nine samples of birch bark used for extraction protocol. The present ratio intensity of these bands for the 1 pp and 3 pp is 5.8 and 6.1 respectively (Additional file 3: Figure S3), showing a higher purity compared to the previously reported extracts [20].

SERS signal of the extract presents large differences in band positions and relative intensity, compared to the corresponding Raman spectrum of the solid extract, confirming the chemisorption of the main specie with respect to the Ag nanoparticles surface. The most enhanced bands were observed in the 1400–1200 cm^-1^ spectral range, where the stretching modes of the skeletal vibrational structure are assigned [21]. The vibrational mode at 1642 cm^-1^ is less enhanced, indicating that the terminal functional group –CH_2_OH of betulin is less involved in the chemisorption on the Ag nanoparticles surface and the betulin structure adopted a more tilted orientation with respect to the Ag nanoparticles. This supposition is confirmed by the broadening of the SERS bands compared to the Raman ones (Additional file 4: Figure S4).

Raman spectroscopy showed that the extracts could be sensitively characterised, allowing to highlight the high betulin content in the solid extracts. SERS spectra revealed a very good reproducibility and stability in time even after 48 hours from sample preparation and also the availability to adsorption on the nanoparticles surface, thus the possibility to detect trace amounts of active compound in aqueous media. Concluding, SERS technique allowed detecting the adsorbed species from natural extract at very low concentrations, compatible with the physiological values. To the best of our knowledge, this is the first SERS report of adsorbed betulin from natural extracts.

**Figure 2:**
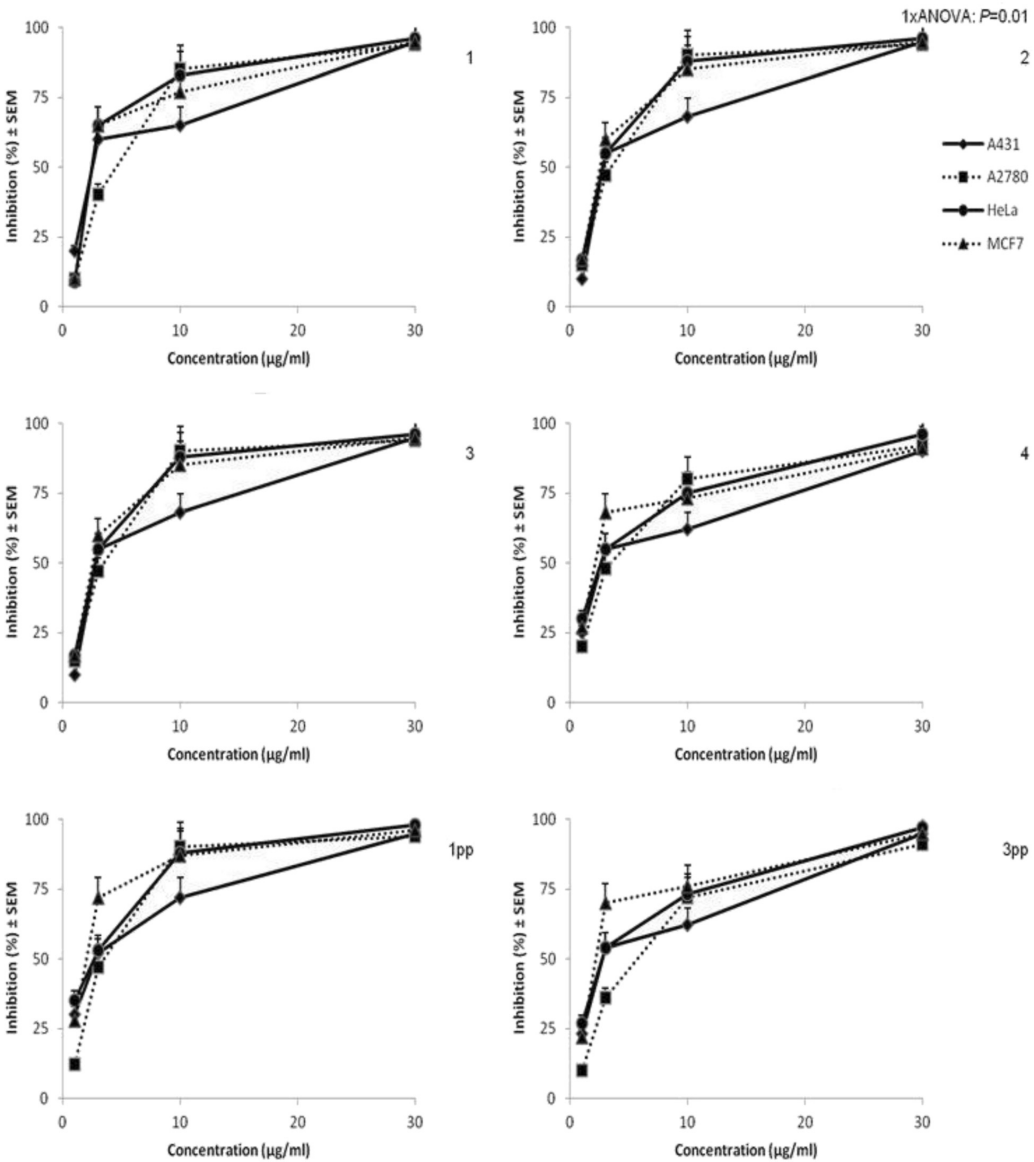
Inhibition of proliferation for birch tree extracts applied on 4 tumor cell-lines (A431, A2780, HeLa and MCF7).

All of the prepared bark extracts exerted a pronounced antiproliferative effect against human cancer cell lines. The currently used four cell lines exhibited similar sensitivities for the extracts and no substantial differences were detected between proliferation inhibitory potencies of the tested samples (Figure 2).

The goal of the MTT assay was a direct comparison of the extracts, concluding that the IC_50_ values of all the samples are between 1 and 5 μg/ml. Therefore, the substantial differences in betulin and betulinic acid content of the extracts are not reflected in the antiproliferative activities. The reason of this contradiction is out of the scope of the present work, but the presence of other active natural compounds in birch bark, including flavonoids, seems like an obvious explanation [22].

Betulin is known as an anti-inflammatory compound [7]. Extracts of birch bark were tested in actinic keratosis, showing an important therapeutic activity [23]. Birch tree outer bark contains betulin as major compound, but also other pentacyclic triterpens [18]. This composition can produce differences in the biological activity of pure betulin and total bark extract. An important intervention in the inflammatory process can be correlated with the antiproliferative activity. Various anti-inflammatory compounds can diminish tumor incidence and metastases development. This aspect was noticed in different experimental animal models [24].

**Figure 3:**
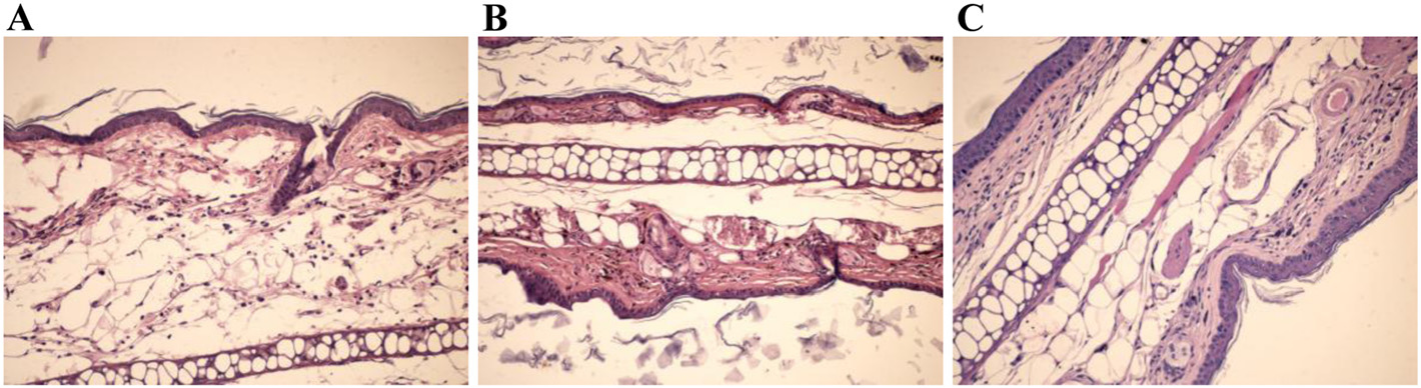
**Test samples: A. TPA, B. indometacin and C. betulin. A.** Epythelial and gristly tissue with a deep and large oedema (HE x 200)-inflammation with croton oil, **B.** Epythelial gristly and muscular tissue with a discreet suephythelial inflammation (HE x 200), **C.** Epythelial gristly and muscular tissue with a discreet oedema, inflammatory subepithelial infiltrate and rare hyperemiate vessels (HE x 200) betulin treatment.

**Figure 4:**
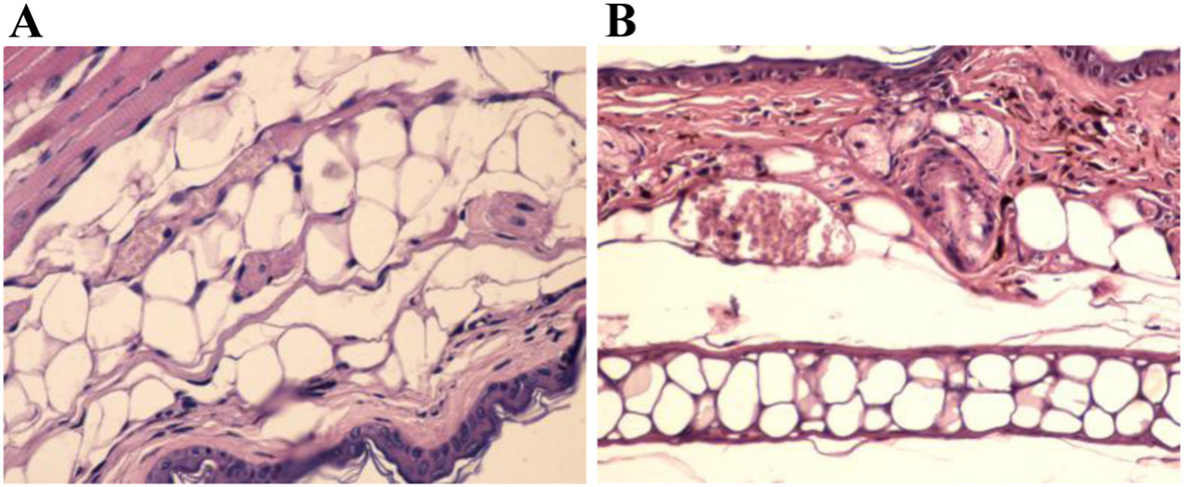
**Treatment with A. 1 pp and B. 3 pp samples.A.** Epythelial and gristly tissue: discreet subepitelial inflammator infiltrate (HE x 200), 1 pp treatment, Epythelial gristly and muscular tissue with a discreet subepitelial inflammation and hiyperemiate blood vessels (HE x 200) 3 pp treatment.

In a few hours TPA can induce an inflammatory process by increasing vascular permeability, producing oedema and swelling inside dermis [25]. Both betulin and 1 pp and 3 pp extract determine an intense reduction of oedema. Indometacin is known as a potent anti-inflammatory drug used as reference for the ear oedema model in our research. Dose evaluation regarding indometacin LD_50_ 50 mg/kg in mice is based on a 14-days mortality response. This dosage is correlated with 1.25 mg indometacin/25 mg mouse, just above the topically administered dose. TPA – ear model is useful for the screening of new topical anti-inflammatory compounds and vegetal extracts. TPA topical application determines mass cell infiltration accompanied by the release of mediators which increase vascular permeability and promote neutrophil influx [25]. TPA topical application also causes the production of oxidative stress that can accelerate local injury [26]. Compounds that inhibit COX or LOX enzymes have also shown the inhibition of TPA-ear inflammation [25] and can be regarded as potent antitumor compounds. Betulin is comparable with indometacin and also the analyzed birch tree extracts in terms of efficacy; however, the extracts reveal the most intense anti-inflammatory potential (Figures 3 and 4), because of the aggregation of effects of other triterpenes, also present in the extract composition, even in small concentrations (betulinic acid , lupeol etc.).

**Figure 5:**
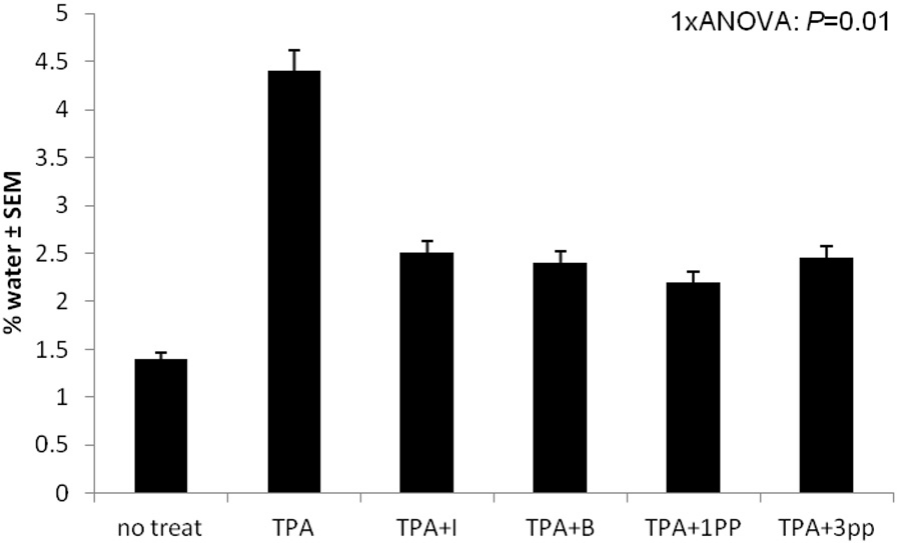
Percentage of water content on ear measurement.

Because of the intense anti-inflammatory activity of 3 pp extract, which shows the highest betulin content, the main conclusion which can be drawn is that betulin is a promising compound in this therapeutic field, in perfect correspondence with other previously reported data [27]. Our work compared betulin to a potent anti-inflammatory agent, indometacin, and the remarkable effects of the pentacyclic triterpene are obvious even after the application of water content evaluation test [26] depicted in Figure 5; this method allowed us to conclude that the anti-inflammatory activity of birch bark extracts is comparable to the one of indometacin.

All the details about betulin extraction from natural sources and also its mechanism of activity increase the interest in the therapeutic application of this promising pharmacological agent.

## Conclusions

New extraction protocol revealing a high betulin content in the outer bark of the birch tree is reported. The extraction products have been extensively characterized using HPLC, NMR, Raman and SERS spectroscopy. Combination between the extraction procedure using solvents and a proper temperature led to a final product with a very high content in active compounds such as betulin. Betulin together with extracts with a high concentration of betulin developped a strong *in vitro* antiproliferative effect and an important *in vivo* activity, mainly anti-inflammatory activity, which adds to its potent antitumor properties.

## Methods

### Plant material

The bark of birch tree (*Betula pendula* Roth, Betulaceae family) was collected in April 2010 from Ponoarele, Mehedinţi, Romania (Source A) and from Aninei Mountains, Romania (Source B). The plant material was authenticated by the Botanical Department, Faculty of Pharmacy, University of Medicine and Pharmacy, Timisoara, Romania, where the voucher specimens (1120 and 1121) have been deposited. The plant material is grey-white and peels in horizontal stripes.

### Extraction of active principles

15 g of birch bark vegetal product (source A and B, see Table 2*) were placed inside a filter paper thimble in a Soxhlet extractor, then extracted with 2-propanol or ethanol (96% v/v) as mentioned in Table 2. The reagents used were commercial products (Reactivul Bucuresti) and used without further purification. Melting points (m.p.) were determined on a Böetius PHMK (Veb Analytik Dresden) instrument. All extracts were standardized and the content in active principles was established.

**Table 2:** Extraction of active principles from the birch bark

**Mass of birch bark (Source)**	**Extraction solvent**	**Aspect of extract**	**Resulting samples**
15 g (A)	2-Propanol (400 mL)	Cream-colored solution + white crystals deposited at the bottom of the flask	The white crystals: **sample 1 pp**
			Evaporation of solution and filtration: **sample 1**
15 g (A)	Ethanol (96% v/v) (400 mL)	Cream-colored solution	Evaporation of solution and filtration: **sample 2**
15 g (B)	2-Propanol (400 mL)	Cream-colored solution + white crystals	The white crystals: **sample 3 pp**
			Evaporation of solution and filtration: **sample 3**
15 g (B)	Ethanol (96% v/v) (400 mL)	Cream-colored solution	Evaporation of solution and filtration: **sample 4**

*Source A: birch bark collected in April 2010 from Ponoarele, Mehedinţi, Romania.

*Source B: birch bark collected in April 2010 from Aninei Mountains, Romania.

### Preparation of dry extracts

After 6 h of extraction, all the flasks were allowed to cool at room temperature (~25°C) for 24 h. For the 2-propanol extracts from both sources A and B, one can notice the spontaneous formation of crystalline precipitates (1 pp and 3 pp, respectively) which were separated by vacuum filtration and washed with cold distilled water. Each precipitate was then dried in an oven at 90°C for 24 h (m.p = 256–258°C for 1 pp and 255-256°C for 3 pp respectively). After their removal, the resulted solution was concentrated to approx. 80 mL using a rotary evaporator (100 mmHg, 50°C), leading to the separation of cream-colored solids (1 and 3, respectively) by vacuum filtration, washed with cold water and then dried in an oven at 90°C for 24 h.

The ethanolic extracts from both sources A and B were concentrated to approx. 80 mL using a rotaevaporator (100 mmHg, 50°C); the resulted cream-colored solids (2 and 4) were separated by vacuum filtration and washed on with cold water, then dried in a oven at 90°C for 24 h.

The dried extracts were weighed and the yield was calculated based on the wet weight of birch bark. The extraction yield varied from 7 to 10% depending on the extraction solvent, the yield in propanol being higher compared to ethanol.

### HPLC analysis

#### HPLC coupled with mass spectrometer

HP 1100 Series binary pump, Degasser online 1100 Agilent, auto sampler HP 1100 Series, thermostat HP 1100 Series, mass spectrometer Agilent Ion Trap 1100 SL

*HPLC working conditions:* Analytical column: Zorbax SB-C18 100 mm x 3.0 mm i.d., 3.5 μm (Agilent), Filter on-line 0.2 microns (Agilent). Mobile phase: isocratic elution using formic acid 0.4% (V/V) / methanol 14/86 (V/V). Flow: 1 ml/min, temperature: 40°C. Injection volume: 2 μl

Detection:

● Betulin - MS – ion m/z 425.6 monitoring, corresponding to protonated betulin dehydration product [M-H_2_O]^+^

● Betulinic acid - MS – ion m/z 439.6 monitoring, corresponding to protonated betulinic acid dehydration product [M-H_2_O]^+^

*MS working conditions:* Ions source: APCI + (atmospheric pressure chemical ionisation). Ionization mode: positive. Electrospray: nitrogen, pressure 60 psi. Vaporiser: 400ºC. Drying gas: nitrogen, debit 7 L/min, temperature 300ºC. Capillary voltage: 2000 V. Analysis mode: SIM-MS, m/z 425.6 (betulin) and 439.6 (betulinic acid)

All chromatographic operations were carried out at ambient temperature.

#### Standards

For quantitative determination betulin / betulinic acid standards were used (Extrasynthese, France, lot 05112416 and lot 05112407, respectively).

#### Sample processing

10 ± 5 mg powder were weighed in a 10 ml flask and solubilised with a mixture of acetonitril / acetone 50/50. Two dilutions were prepared: 1/1000 for betulin analysis and 1/100 for betulinic acid.

Calibration curve for betulin and betulinic acid were built within the concentration range of 44.2-884 ng/ml and 39.5-790 ng/ml, respectively (Additional file 5: Table S1 and Additional file 6: Table S2, Additional file 7: Figures S5 and Additional file 8: Figure S6). All solutions were prepared in methanol / water 50/50.

### NMR Spectroscopy

The carbon spectra were recorded at 150,86 MHz ^13^C Larmor frequency with a Bruker AVANCE-600 spectrometer, using the standard CP/MAS pulse sequence [28]. The spinning frequency of the sample was ν_R_ = 12 kHz, the applied ^1^H 90° pulse length was 3.8 μs and the signal was acquired under two-pulse phase-modulated (TPPM) [29]^1^H decoupling at 70 kHz by averaging 10.000 scans with a recycle delay of 3 s. The CP transfer was optimized for the first Hartmann – Hahn matching condition (ν_1C_ = ν_1H_ - ν_R_) and a contact pulse of 1.5 ms was used. All the experiments were performed at room temperature. Carbon-13 chemical shifts are expressed in parts per million (ppm) and calibrated with respect to tetramethylsilane.

### Raman and SERS spectroscopy

FT-Raman spectra of the extracts have been recorded using a Brucker Equinox 55 spectrometer with an integrated FRA 106 s Raman module fiber optic coupled with the Ramanscope II Raman microscope. The 1064 nm line from a Nd: YAG laser has been employed for Raman excitation and a Ge detector operation at liquid nitrogen temperature for detection.

Additionally, surface enhanced Raman scattering (SERS) has been employed in order to get insight into the adsorption properties of the extract compounds. Citrate reduced Ag colloidal nanoparticles have been employed as SERS substrate. The localised surface plasmon resonance (LSPR) of such nanoparticles exhibits an electronic absorption band at 410 nm. 3.8 mg extract 1 pp has been dissolved in 2 ml ethanol and sonicated for 10 minutes. Upon adding to Ag nanoparticles trace amount (cca 10 ul) of ethanol-extract solution, the Ag colloid changes in colour from grey-yellow to dark, suggesting a complementary aggregation in the presence of the adsorbed species. Consequently, their LSPR shifts to higher wavelength, therefore the 632 nm laser line excitation falls within the resonance condition of the so-called Ag-adsorbate charge transfer band and an enhanced Raman signal of the Ag-betulin complex could be obtained. SERS spectra have been recorded using a DeltaNU Raman spectrometer equipped with a He-Ne laser emitting at 632.8 nm. The laser power was 40 mW and the spectral resolution of 10 cm^-1^.

### Determination of antiproliferative activities

Growth inhibiting effects were measured *in vitro* on four malignant human cell lines (ECACC, Salisbury, UK): A431 (skin epidermoid carcinoma), A2780 (ovarian carcinoma), HeLa (cervix adenocarcinoma) and MCF7 (breast adenocarcinoma) by means of MTT assay [30]. The cells were maintained in minimal essential medium (Sigma-Aldrich) supplemented with 10% fetal bovine serum, 1% non-essential amino acids and an antibiotic-antimycotic mixture. Briefly, near-confluent cancer cells were seeded onto a 96-well microplate (5000 cells/well) and, after an overnight new medium (200 μL) containing the tested extracts was added. The highest concentration was 30 μg/ml. After incubation for 72 h at 37°C in humidified air containing 5% CO_2_, the living cells were assayed by the addition of 5 mg/mL MTT solution (20 μL). MTT was converted by intact mitochondrial reductase and precipitated as blue crystals during a 4-h contact period. The medium was then removed and the precipitated formazan crystals were dissolved in DMSO (100 μL) during a 60-min period of shaking at 25°C. Finally, the reduced MTT was assayed at 545 nm, using a microplate reader; wells with untreated cells were utilized as controls. Stock solutions of the tested substances (10 mg/ml) were prepared with DMSO. The highest DMSO content of the medium did not have any significant effect on the cell proliferation.

### Animal studies

Animal studies were conducted on C57BL/6 J female mice of 8 weeks old. Mice were purchased from Charles River (Sulzfeld, Germany). The work protocol followed all NIAH-National Institute of Animal Health rules: animals were maintained during the experiment in standard conditions: 12 h light–dark cycle, food and water ad libitum, temperature 24°C, humidity above 55%. All experiments were approved by UMFVBT Bioethical Committee. The number of mice taken into study was twenty four divided in six groups as follows:

● group A : mice on which TPA in acetone was administrated on the ear

● group B: mice on which TPA in acetone was administrated on the ear followed after 30 minutes by indomethacin as control

● group C: mice on which TPA in acetone was administrated on the ear followed after 30 minutes by betulin

● group D: mice on which TPA in acetone was administrated on the ear followed after 30 minutes by 1 pp extract

● group E mice on which TPA in acetone was administrated on the ear followed after 30 minutes by 3 pp extract

● group I: blank (non-treated)

Inflammation was induced in both ears of each mouse by the topical application of 2 μg TPA dissolved in 20 μL of acetone to both the inner and outer ear surfaces. Thirty minutes after the application of TPA, the inner and outer surface of each ear was treated with 2 mg of betulin, or the equivalent quantity of birch tree extract. The same quantity of indomethacin, 2 mg, was used as positive control. The solvent was acetone: DMSO in molar ratio 9:1 and the quantity administrated was 0,1 ml [25,31]. Mice were killed after 4 h by cervical dislocation and ears were collected. The ears were weighted immediately and considered as wet weight. The ears were dried for 24 h in dry oven, weighted again and the weight defined as dry weight. Skin water content was calculated by subtracting the dry weight from the wet weight and then dividing to wet weight. It is expressed as H_2_O/mg dry weight [26].

For the histological analysis, tissue samples (skin) were fixed in 10% formalin solution, embedded in paraffin and cut at 4 microns. Finally, after removal of paraffine, the samples were stained with H&E (hematoxylin-eosin) and microscopically analyzed.

Experiments were carried out in triplicate and statistically analyzed by one-way ANOVA and presented as mean ± SEM; p < 0.01 was considered significant.

## Competing interests

The authors declare no conflict of interest.

## Authors ’ contributions

CS, IL and OV carried out the extraction procedures. CS, MA, MM and SCP carried out the physical-chemical analysis of the extracts. CD, AG and IZ carried out the biological tests. All authors participated in the article’s design and coordination and helped to draft the manuscript. All authors read and approved the final manuscript.

## Supplementary Material

Additional file 1 **Figure S1.** Full-scan spectra of betulin (above) and betulinic acid (below) in the mobile phase. Click here for file

Additional file 2 **Figure S2.** Solid-state NMR ^13^C spectrum of the 3 pp sample. Click here for file

Additional file 3 **Figure S3.** FT-Raman spectra of the 1 pp and 3 pp extracts (c and d, respectively) in comparison with the spectra of betulin (a) or early reported extract (b) (Dehelean et. al, JOAM, 2007 [20]). Note the relative intensity of the band marked with arrow, showing the impurity presence. Excitation 1064 nm, 350 mW. Click here for file

Additional file 4 **Figure S4.** SERS spectra of the sample 1 (a and b) in comparison with the FT-Raman of the solid 1 pp sample. The solvent (ethanol) spectrum (d) is also given to avoid misinterpretation of the Raman bulk contribution to the overall spectral shape. Excitation: 632.8 nm (a, b, d) and 1064 nm, c). Click here for file

Additional file 5 **Table S1.** Concentrations used for betulin calibration curve. Click here for file

Additional file 6 **Table S2.** Concentrations used to built calibration curve of betulinic acid. Click here for file

Additional file 7 **Figure S5.** Betulin calibration curve. Click here for file

Additional file 8 **Figure S6.** Betulinic acid calibration curve. Click here for file
