# Supplementary material for: Study of the betulin enriched birch bark extracts effects on human carcinoma cells and ear inflammation
Source: Chem Cent J. 2012 Nov 19;6:137. doi: 10.1186/1752-153X-6-137 (PMC3527166; doi:10.1186/1752-153X-6-137)
Supplement: Additional file 2 — Figure S2. Solid-state NMR 13C spectrum of the 3 pp sample. [file 1752-153X-6-137-S2.doc]

**Figure 2S.**

**Solid-state NMR ^13^C spectrum of the 3pp sample.**
