# Supplementary material for: Study of the betulin enriched birch bark extracts effects on human carcinoma cells and ear inflammation
Source: Chem Cent J. 2012 Nov 19;6:137. doi: 10.1186/1752-153X-6-137 (PMC3527166; doi:10.1186/1752-153X-6-137)
Supplement: Additional file 3 — Figure S3. FT-Raman spectra of the 1 pp and 3 pp extracts (c and d, respectively) in comparison with the spectra of betulin (a) or early reported extract (b) (Dehelean et. al, JOAM, 2007 [20]). Note the relative intensity of the band marked with arrow, showing the impurity presence. Excitation 1064 nm, 350 mW. [file 1752-153X-6-137-S3.doc]

**Figure 3S.**

**FT-Raman spectra of the 1pp and 3pp extracts (c and d, respectively) in comparison with the spectra of betulin (a) or early reported extract (b) (Dehelean et. al, JOAM, 2007 [20]). Note the relative intensity of the band marked with arrow, showing the impurity presence. Excitation 1064 nm, 350 mW.**
