# Supplementary material for: Study of the betulin enriched birch bark extracts effects on human carcinoma cells and ear inflammation
Source: Chem Cent J. 2012 Nov 19;6:137. doi: 10.1186/1752-153X-6-137 (PMC3527166; doi:10.1186/1752-153X-6-137)
Supplement: Additional file 4 — Figure S4. SERS spectra of the sample 1 (a and b) in comparison with the FT-Raman of the solid 1 pp sample. The solvent (ethanol) spectrum (d) is also given to avoid misinterpretation of the Raman bulk contribution to the overall spectral shape. Excitation: 632.8 nm (a, b, d) and 1064 nm, c). [file 1752-153X-6-137-S4.doc]

**Figure 4S.**

**SERS spectra of the sample 1 (a and b) in comparison with the FT-Raman of the solid 1pp sample. The solvent (ethanol) spectrum (d) is also given to avoid misinterpretation of the Raman bulk contribution to the overall spectral shape. Excitation: 632.8 nm (a, b, d) and 1064 nm, c).**
