# Supplementary material for: Study of the betulin enriched birch bark extracts effects on human carcinoma cells and ear inflammation
Source: Chem Cent J. 2012 Nov 19;6:137. doi: 10.1186/1752-153X-6-137 (PMC3527166; doi:10.1186/1752-153X-6-137)
Supplement: Additional file 5 — Table S1. Concentrations used for betulin calibration curve. [file 1752-153X-6-137-S5.doc]

Table 1S. Concentrations used for betulin calibration curve

| Theoretical concentration (ng/ml) | Measured concentration (ng/ml) | Accuracy (%) |
| --- | --- | --- |
| 44.20 | 45.54 | 103.04 |
| 88.40 | 84.21 | 95.26 |
| 176.80 | 167.78 | 94.90 |
| 353.60 | 367.76 | 104.00 |
| 442.00 | 470.39 | 106.42 |
| 663.00 | 654.95 | 98.79 |
| 884.00 | 883.70 | 99.97 |
