# Supplementary material for: Study of the betulin enriched birch bark extracts effects on human carcinoma cells and ear inflammation
Source: Chem Cent J. 2012 Nov 19;6:137. doi: 10.1186/1752-153X-6-137 (PMC3527166; doi:10.1186/1752-153X-6-137)
Supplement: Additional file 6 — Table S2. Concentrations used to built calibration curve of betulinic acid. [file 1752-153X-6-137-S6.doc]

Table 2S. Concentrations used to built calibration curve of betulinic acid

| Theoretical concentration (ng/ml) | Measured concentration (ng/ml) | Accuracy (%) |
| --- | --- | --- |
| 39.50 | 41.85 | 105.96 |
| 79.00 | 71.79 | 90.87 |
| 158.00 | 159.09 | 100.69 |
| 316.00 | 328.70 | 104.02 |
| 395.00 | 394.35 | 99.83 |
| 592.50 | 576.39 | 97.36 |
| 790.00 | 797.33 | 100.93 |
