# Supplementary material for: Study of the betulin enriched birch bark extracts effects on human carcinoma cells and ear inflammation
Source: Chem Cent J. 2012 Nov 19;6:137. doi: 10.1186/1752-153X-6-137 (PMC3527166; doi:10.1186/1752-153X-6-137)
Supplement: Additional file 7 — Figure S5. Betulin calibration curve. [file 1752-153X-6-137-S7.doc]

**Figure 5S. Betulin calibration curve.**
